# Advances in optical gastrointestinal endoscopy: a technical review

**DOI:** 10.1002/1878-0261.12792

**Published:** 2020-09-19

**Authors:** Yubo Tang, Sharmila Anandasabapathy, Rebecca Richards‐Kortum

**Affiliations:** ^1^ Department of Bioengineering Rice University Houston TX USA; ^2^ Division of Gastroenterology Baylor College of Medicine Houston TX USA

**Keywords:** gastrointestinal tract, machine learning, molecular probe, optical endoscopy

## Abstract

Optical endoscopy is the primary diagnostic and therapeutic tool for management of gastrointestinal (GI) malignancies. Most GI neoplasms arise from precancerous lesions; thus, technical innovations to improve detection and diagnosis of precancerous lesions and early cancers play a pivotal role in improving outcomes. Over the last few decades, the field of GI endoscopy has witnessed enormous and focused efforts to develop and translate accurate, user‐friendly, and minimally invasive optical imaging modalities. From a technical point of view, a wide range of novel optical techniques is now available to probe different aspects of light–tissue interaction at macroscopic and microscopic scales, complementing white light endoscopy. Most of these new modalities have been successfully validated and translated to routine clinical practice. Herein, we provide a technical review of the current status of existing and promising new optical endoscopic imaging technologies for GI cancer screening and surveillance. We summarize the underlying principles of light–tissue interaction, the imaging performance at different scales, and highlight what is known about clinical applicability and effectiveness. Furthermore, we discuss recent discovery and translation of novel molecular probes that have shown promise to augment endoscopists' ability to diagnose GI lesions with high specificity. We also review and discuss the role and potential clinical integration of artificial intelligence‐based algorithms to provide decision support in real time. Finally, we provide perspectives on future technology development and its potential to transform endoscopic GI cancer detection and diagnosis.

## Graphical abstract

We provide a technical review of optical endoscopic imaging technologies for GI cancer screening and surveillance. This review summarizes the principles of light–tissue interaction, the imaging performance at different scales, and highlights what is known about clinical applicability and effectiveness. Furthermore, we discuss the translation of novel molecular probes and integration of artificial intelligence‐based algorithms to provide decision support.

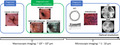

**Abbreviations**
ADR: adenoma detection rate
AI: artificial intelligence
ASGE: American Society for Gastrointestinal Endoscopy
CADe: computer‐aided detection
CADx: computer‐aided diagnosis
CE: chromoendoscopy
CLE: confocal laser endomicroscopy
EAC: esophageal adenocarcinoma
ESCC: esophageal squamous cell carcinoma
ESGE: European Society of Gastrointestinal Endoscopy
FICE: Fuji Intelligent Chromo Endoscopy
FOV: field of view
GI: gastrointestinal
HRME: high‐resolution microendoscope
NBI: narrowband imaging
PIVI: preservation and incorporation of valuable endoscopic innovations
RCT: randomized controlled trials
SECM: spectrally encoded confocal endomicroscopy
VLE: volumetric laser endomicroscopy
WLE: white light endoscopy

## 1 Introduction

Cancers in the gastrointestinal (GI) tract, including the esophagus, stomach, small intestine, and colon, are among the 10 most common cancers worldwide, imposing a significant healthcare burden globally [1]. Carcinogenesis in the GI tract typically involves a cascade of molecular dysregulation and architectural alternations; thus, a significant proportion of the resulting morbidity, mortality, and healthcare cost can potentially be prevented through detection and treatment of precursor lesions and early cancers. To detect cancer at early stages, a combination of screening and surveillance programs, including both endoscopic and nonendoscopic tests, have been developed and implemented. Recommended screening and surveillance programs vary by population risk and geography.

Current strategies for early detection of esophageal neoplasia depend on the type of esophageal cancer which is most prevalent. Globally, 90% of esophageal cancers are categorized as esophageal squamous cell carcinoma (ESCC); ESCC is most prevalent in certain geographical regions such as East Asia, Iran, and Africa [1, 2]. In these regions, screening endoscopy in conjunction with Lugol's chromoendoscopy (CE) is advocated as the gold standard modality based on its high sensitivity [3, 4], although limited specificity remains a challenge. In Western countries, esophageal adenocarcinoma (EAC) is the predominant histologic subtype. Population‐based screening endoscopy is not currently recommended due to the low incidence. However, the last few decades have witnessed dramatically increasing rates of incidence of EAC [5], and guidelines in the United States and Europe recommend white light endoscopic surveillance of patient with Barrett's esophagus, the only known precursor to EAC [6, 7]. Nonetheless, conventional white light endoscopy (WLE) is found to frequently miss early cancers in BE [8]. In addition to standard endoscopy, more affordable and less invasive endoscopic and tissue sampling approaches, such as ultrathin endoscopy and Cytosponge combined with biomarkers [9], are also under evaluation as alternative methods for esophageal cancer screening.

For gastric cancer, countries with a high incidence such as Japan and South Korea have implemented national screening programs [1, 10, 11]. While less invasive nonendoscopic screening methods such as barium upper GI series can be used, endoscopy remains the primary tool in high incidence countries with higher cancer detection rates and biopsy capability [12, 13]. In regions with a low or intermediate incidence, endoscopy is only recommended for individuals at an increased risk for gastric cancer [14, 15].

Colorectal cancer is the third most common cancer globally and ranks second in mortality [1]. Adenomatous polyps are the most important precursor lesions for colorectal cancer, and their detection and removal through polypectomy are associated with reduced cancer incidence and morbidity [16, 17]. Large‐scale screening is commonly practiced in North American and European countries [18], offering a range of nonendoscopic and endoscopic screening tests, including guaiac‐based fecal occult blood test, fecal immunochemical test, sigmoidoscopy, and colonoscopy. The clinical adoption of these modalities varies in different countries [19], but colonoscopy is the only gold standard screening tool that offers direct visualization, same‐session detection, and removal of polyps across the entire colon. With recent advances in optical endoscopy, there is a significant interest in optical diagnosis of diminutive (≤ 5 mm) colorectal polyps that represent a vast majority of all polyps, yet are only linked with minimal risks of malignant progression [20].

**Fig. 1:**
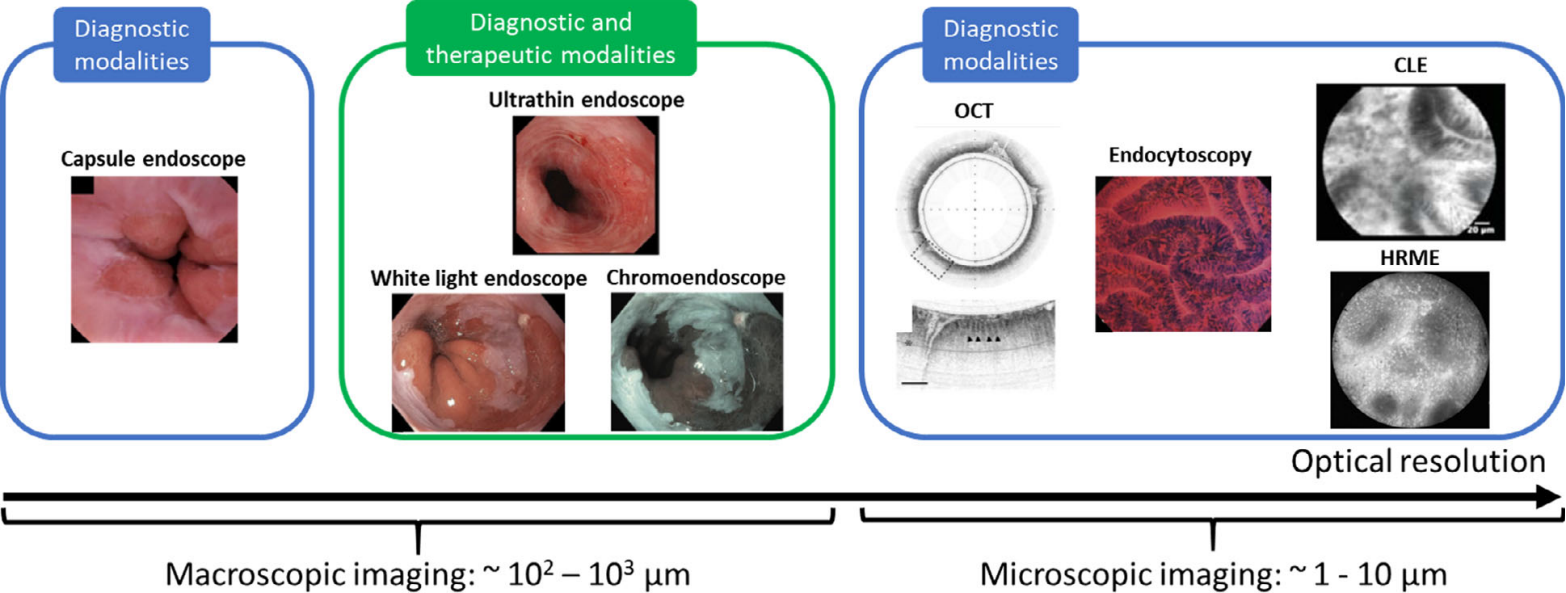
Optical endoscopic techniques for macroscopic and microscopic imaging of the GI mucosa. Existing macroscopic modalities include high‐definition endoscopy, ultrathin endoscopy, and capsule endoscopy. Microscopic resolution can be achieved using OCT, endocytoscopy, CLE, and HRME. Reproduced from [66, 81, 136] with permission from Elsevier (white light and chromoendoscope, capsule endoscope, and endocytoscopy, respectively), from [55] with permission from John Wiley and Sons (ultrathin endoscope), from [137] by permission from Springer Nature (OCT), from [69] with permission from © Georg Thieme Verlag KG (CLE).

Currently, standard endoscopes remain the primary diagnostic and therapeutic tool for GI cancer screening and surveillance (Fig. 1). Standard endoscopy, in which either upper or lower GI tract of a sedated patient is thoroughly examined with a white light endoscope by a trained endoscopist, allows for imaging, biopsies, and treatment in a single endoscopic session. However, despite its central role, many studies report that standard endoscopy frequently misses GI lesions at early stages [8, 21, 22], primarily due to the inability to visualize subtle architectural changes under conventional white light illumination. The development of novel endoscopic technologies with improved accuracy, enhanced sampling, and minimal invasiveness could play a pivotal role to advance early detection and clinical management of GI lesions. Figure 1 highlights existing and novel endoscopic technologies that offer multimodal imaging of GI lesions to improve early detection at macroscopic and microscopic scales. Overall, macroscopic modalities can interrogate a large field of view (FOV) and serve as “red‐flag” techniques to rapidly survey the entire lumen and identify suspicious lesions. In a two‐step protocol, modalities with microscopic resolution can be used to further examine suspicious lesions with cellular or subcellular detail (10 µm or higher). As a minimally invasive macroscopic modality, capsule endoscopy offers an appealing option for “red‐flag” imaging. Alternatively, high‐resolution endoscopic modalities such as confocal laser endomicroscopy (CLE) and endocytoscopy can provide histologic information in real time.

Together, this wide range of technologies in Fig. 1 provides powerful tools for endoscopists to examine the GI tract both macroscopically and microscopically, forming the basis for the next generation of GI endoscopy. As summarized in Table 1, to help guide translation of promising new technologies, the American Society for GI Endoscopy (ASGE) created the preservation and incorporation of valuable endoscopic innovations (PIVI) performance thresholds that new technologies for assessment of Barrett's esophagus and colorectal polyps should meet prior to adoption [23, 24].

**Table 1:** PIVI performance thresholds to adopt new imaging technologies for GI lesion assessment [23, 24].

Clinical condition	Imaging‐guided endoscopic management	Required performance thresholds
Barrett's esophagus	Perform targeted biopsies (without random biopsies)	For diagnosis of HGD and EAC Sensitivity > 90% and specificity > 80% Negative predictive value (NPV) > 98%
Rectosigmoid polyps	Leave suspected hyperplastic polyps 5 mm or smaller without resection	For diagnosis of adenomatous histology NPV > 90%, when used with high confidence
Colorectal polyps	Resect and discard polyps 5 mm or smaller without histopathology evaluation	For determining postpolypectomy surveillance intervals > 90% agreement with histopathology, when used with high confidence and in combination with histopathology evaluation of polyps > 5 mm

**Fig. 2:**
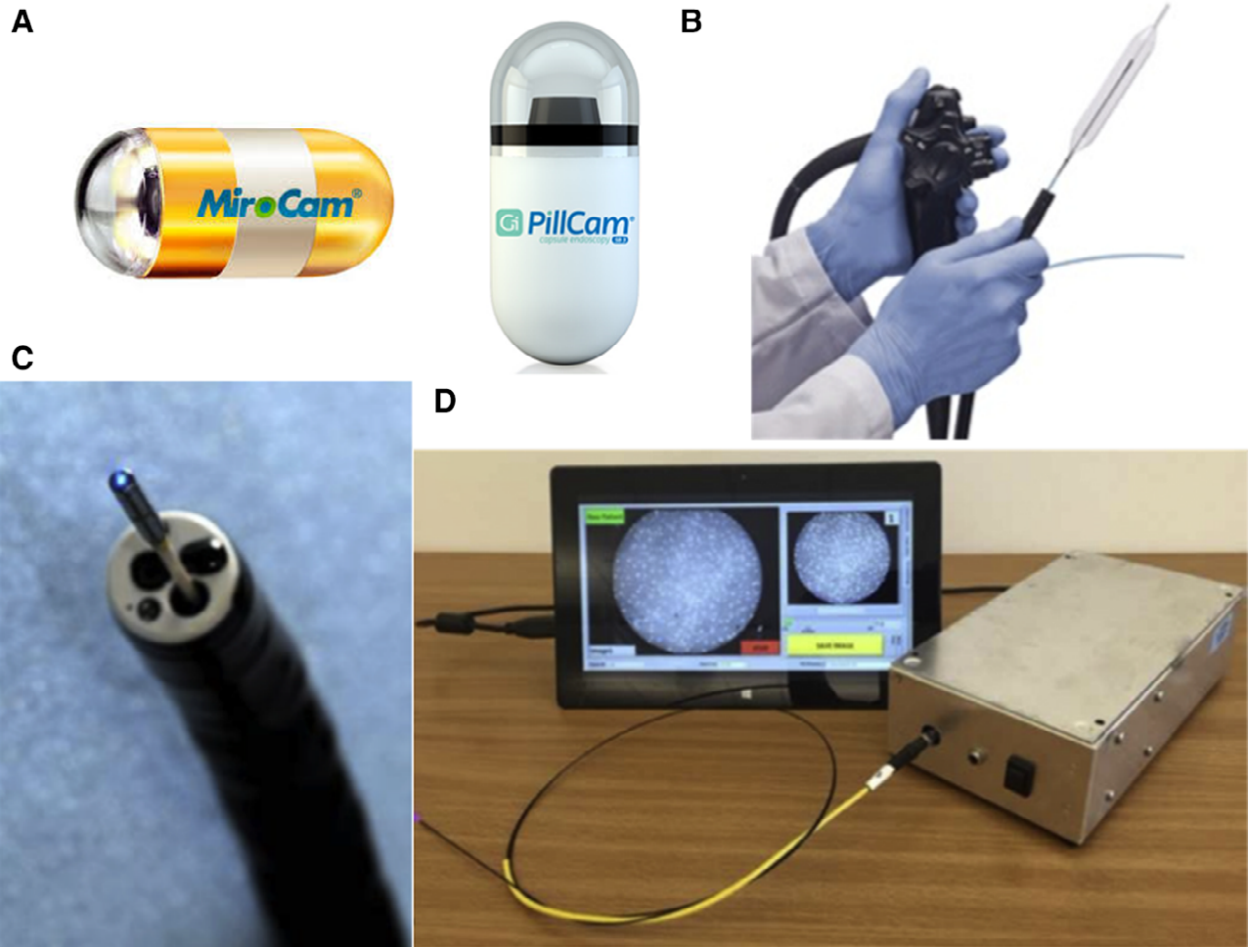
Examples of capsule‐, balloon‐, and probe‐based optical endoscopic systems. (A) Capsule endoscopes (MicroCam and PillCam). (B) VLE probe within an inflated balloon catheter. (C) Confocal laser endomicroscope through a biopsy channel. (D) A low‐cost HRME with integrated diagnostic software. Figure 2B–D reproduced from [97, 138, 139] with permission from Elsevier.

In this technical review, we provide an overview of the current status of existing and emerging optical imaging modalities for GI endoscopy. Previous reviews of endoscopic imaging techniques in the GI tract have been provided by the ASGE technology committee, the European Society of GI Endoscopy (ESGE) research committee, and other authors [18, 24, 25, 26], with a strong focus on the performance of commercially available and commonly studied modalities such as CE and CLE, while providing detailed descriptions of available evidence of their clinical performance. In this review, we focus on the technical aspects and clinical applicability of optical endoscopy, highlighting recent advances in device and optical design, molecular probes, and machine‐learning algorithms for image interpretation. In addition to commercially available platforms, we also discuss emerging technologies at earlier stages of clinical translation, summarizing how they exploit different dimensions of light–tissue interaction to identify lesions at early stages of GI cancer progression. For each modality, we discuss the imaging capabilities while highlighting the fundamental principles of light–tissue interactions and their implications for clinical usefulness. We review imaging systems with various novel form factors (examples in Fig. 2), including capsules, balloon catheters, and probes, and discuss how they can contribute to multimodal and less invasive imaging of the GI tract with enhanced contrast and resolution. We also review the clinical translation and integration of novel molecular probes and machine‐learning algorithms.

## 2 Macroscopic imaging systems

### 2.1 Current standard of care: white light endoscopy

Because of the unique anatomy of the GI tract, conventional endoscopy with white light illumination remains the gold standard to assess GI lesions. Despite its routine use, there is a critical need to further improve the diagnostic performance of WLE. For surveillance of Barrett's esophagus, for example, four‐quadrant random biopsy sampling (referred to as the Seattle protocol) remains an important and essential component of current guidelines [29]. Similarly, CE using Lugol's staining is generally practiced for ESCC screening in many Asian countries. In screening colonoscopy, the use of high‐definition endoscopy with advanced modalities such as virtual CE has also been recommended by the ESGE [18].

Over the last few decades, the technical performance of WLE has benefited from improvements in imaging sensors and optics that offer greater pixel density and higher magnification. Since the late 1990s, high‐definition endoscopes have become widely available, enabling more meticulous examination of mucosal patterns, and replacing standard‐definition endoscopes as the current modality of choice. Standard high‐definition endoscopy has been further complemented by less invasive ultrathin endoscopes and capsule endoscopes, and the detailed imaging features and specifications are compared in Table 2. Recent commercial systems are also augmented by advanced imaging features, including magnification endoscopes with up to 150‐fold optical magnification and various techniques to enhance mucosal features such as CE. While data comparing high‐definition with standard‐definition WLE are relatively scarce, high‐definition endoscopes have been used in numerous clinical studies, especially in tandem with virtual or dye‐based CE.

**Table 2:** Commercially available endoscopic systems for macroscopic imaging of GI mucosa [140, 141, 142].

Endoscope systems	High‐definition endoscope	Ultrathin endoscope	Capsule endoscope
Endoscope diameter (Approx.)	9–13 mm	5–6 mm	11 mm
FOV	140° to 170°	120° to 140°	145° to 170°
Camera resolution	High definition (up to 2 million pixels)	Standard definition (100 000–400 000 pixels)	256 × 256 to 512 × 512 pixels
Scope guidance	4‐way angulation	2‐way or 4‐way angulation	Passive peristalsis; External magnetic steering
Advanced imaging capability	Yes	Yes	No
Sedation requirement	Yes	No	No
GI tract accessibility	Upper or lower GI	Upper GI	Upper or lower GI, including small bowel
Biopsy capability	Yes	Supported in most models except disposable versions	No

Future development of high‐definition WLE can benefit from further improvements in the optical FOV and resolution, as well as 3D imaging capability. As shown in Table 2, current high‐definition endoscopes can support up to 2K video acquisition with an angular FOV of 140–170°. To better survey the GI anatomy, wide FOV endoscopes are being developed to provide an extra wide angular or near‐panoramic view (245–330°), and preliminary studies have shown their utility for better colon polyp detection and improved visualization of occult regions in the upper GI tract [30, 31, 32, 33]. In addition, endoscopes with UHD resolution (4k or 8k vs. current 2k) and 3D imaging capabilities, although mostly demonstrated in laparoscopic applications or animal models [34, 35, 36], also have the potential to enable more detailed mucosal examination and enhance surgical maneuverability in the GI tract.

### 2.2 Virtual chromoendoscopy

In contrast to WLE based on the entire visible spectrum, virtual CE exploits spectral variations in the interaction of light with tissue to highlight mucosal features, such as blood vessels or changes in light scattering. Because spectral imaging can be achieved by either optical filtering or postprocessing without modifying the imaging optics, virtual CE is seamlessly integrated in most current endoscopic systems. At the touch of a button, it provides a convenient means for endoscopists to investigate lesions with enhanced contrast. First‐generation virtual CE include narrowband imaging (NBI; Olympus, Tokyo, Japan), Fuji Intelligent Chromo Endoscopy (FICE; Fujinon, Tokyo, Japan), and iScan (Pentax, Tokyo, Japan). Newer modalities, such as blue laser imaging, have also been recently introduced. As summarized in Table 3, NBI enhances the contrast of mucosal features and microvascular networks by illuminating the GI surface with blue (415 nm) and green light (540 nm); FICE and iScan, in comparison, are based on postprocessing algorithms to improve vessel visualization and tissue type differentiation. Among these modalities, NBI is the most commonly studied virtual CE modality; thanks to its wide availability, as well as established interpretation criteria with substantial interobserver agreement [37], the modality is well accepted, especially among experts.

**Table 3:** Advanced imaging modalities available in commercial endoscopic platforms.

Imaging modality	Virtual CE			Dye‐based CE			
	NBI	FICE	iScan	Indigo carmine	Methylene blue	Acetic acid	Lugol's iodine
Source of contrast	Reflectance; hemoglobin absorption	Reflectance	Reflectance	Reflectance of exogenous dyes	Absorption by small intestine and colonic epithelium	Acetic whitening	Absorption by tissue with high glycogen
Targeted clinical features	Mucosal patterns and vascular network	Mucosal patterns and vascular network	Mucosal patterns and vascular network	Mucosal topology such as pits and ridges	Uptake by intestinal epithelium	Mucosal patterns	Uptake by normal squamous epithelium

In Barrett's esophagus, CE was shown to improve diagnostic yield for detection of dysplasia/cancer by 34% in a meta‐analysis of 14 studies involving 843 patients [38]. As a widely studied modality, NBI was shown to meet the PIVI thresholds for BE surveillance (Table 1) and its conjunction use with standard WLE is recommended [25, 29]. For gastric precancerous histology, NBI is recommended by the ESGE due to its capability to significantly improve intestinal metaplasia detection compared to high‐definition endoscopy alone [15]. In screening colonoscopy, a meta‐analysis by ASGE reported a 91% NPV using NBI for detection of adenomas in academic centers, surpassing the PIVI threshold of 90% to support a “diagnosis‐and‐leave” strategy for diminutive polyps [24]. However, it should be noted that subpar results that fell short of meeting the same criteria were reported in community settings [39, 40]. Per ESGE recommendations, the use of virtual CE to provide optical diagnosis is only suggested when endoscopy is adequately documented and performed by experts [18].

In settings where the most recent platforms are available, virtual CE has shown great promise to better visualize GI lesions without incurring additional cost and applying exogenous dyes. As most high‐definition endoscope systems offer CE compatibility nowadays, it is gaining momentum and more popularity, especially among experts. To endorse their routine use, however, universal classification criteria need to be established and externally validated, especially among novice users outside of research centers. In addition, associated learning curves and interobserver reliability need to be studied. At the meantime, the importance of technical advances should be recognized. A very recent meta‐analysis of 11 randomized controlled trials (RCTs) revealed that the second‐generation and brighter NBI significantly increased the adenoma detection rate (ADR) compared to WLE, as well as to first‐generation NBI [41].

As an extension to commercially available virtual CE that exploits tissue response in specific spectral bands, multispectral or hyperspectral imaging has also shown value for GI lesion characterization with increased spectral dimensions [42, 43]. Recently, real‐time hyperspectral imaging has been enabled in endoscopic systems and initial clinical evaluation in the GI tract has been reported [44, 45, 46]. Taken together, it is expected that GI lesion detection will be further improved by virtual CE as the technology continually improves and the clinical use becomes standardized.

### 2.3 Dye‐based chromoendoscopy

Unlike optical or computational filtering in virtual CE, dye‐based CE takes advantage of exogenous dyes to enhance contrast of mucosal features, especially in lesions that may appear subtle, flat, or depressed under WLE. In general, two types of dyes are clinically used: absorptive dyes such as methylene blue and Lugol's iodine, and contrast stains such as indigo carmine (Table 3). Among those dyes, Lugol's iodine selectively binds to glycogen that is more abundantly stored in normal than dysplastic squamous epithelium. Methylene blue is preferentially absorbed by epithelial cells of small intestine or colon types, and indigo carmine highlights mucosal topology by filling crevices, pits, and ridges.

The source of imaging contrast, and thus the targeted mucosal features, depends on the staining or uptake mechanisms of specific dyes. Thanks to its high uptake by glycogen‐abundant cells, Lugol's iodine is routinely used as a highly sensitive (92–100% sensitivity), yet inexpensive dye for ESCC screening [2]. For surveillance of Barrett's esophagus, systematic reviews showed improved diagnostic yield and accuracy using acetic acid or methylene blue; therefore, CE is recommended as an adjunctive tool in addition to WLE [29]. For the choice of dyes, acetic acid is inexpensive and meets the ASGE threshold for BE surveillance (Table 1), with early evidence showing that it is more cost‐effective than random biopsies in a high‐risk population [29, 47]. Methylene blue also provides substantial contrast enhancement, but concerns have been raised due to its link to DNA damage [48]. In the stomach, a meta‐analysis of 10 studies using different dyes also demonstrated that dye‐based CE can better detect early gastric cancers and precursors (pooled sensitivity and specificity of 0.90 and 0.82 in 699 patients) than WLE [49].

In settings that lack access to up‐to‐date endoscopic platforms with virtual CE, dye‐based CE offers a safe and relatively inexpensive alternative to enhance mucosal contrast, even though it requires a more cumbersome procedure and relies on the quality and uniformity of the spraying. Like virtual CE, to make the best use of dye‐based CE, user expertise is critical. In addition, a consensus on validated interpretation criteria is yet to be established, which can be particularly challenging given the wide range of dyes and their different uptake or staining patterns.

### 2.4 Ultrathin endoscopy

Compared to standard endoscopes, ultrathin endoscopes are designed to access smaller luminal organs so that endoscopy can be performed during unsedated, outpatient procedures, even though expertise is still required for image interpretation. Thanks to a smaller diameter (6 mm or less, compared to up to 13 mm in standard endoscopes), patients have a higher acceptance and suffer from lower risks of complications and recovery time [50]. The trade‐off includes decreased pixel resolution and reduced mechanical maneuverability (Table 2); a pediatric biopsy forceps can be passed through the accessory channel to obtain biopsies, but the ability to perform more complicated surgical procedures is limited. To further facilitate clinical adoption of the technology, less costly, portable, and disposable versions of ultrathin endoscopes have also been developed, but biopsy capability is not supported [51].

The capability of ultrathin endoscopy to investigate upper GI lesions is reported in several pilot studies. As a less costly outpatient procedure, transnasal endoscopy using ultrathin endoscopes is considered a potential alternative for BE screening [7], even though its role in BE surveillance is limited by the relatively low imaging quality. In a pilot randomized cross‐over study involving 82 patients, transnasal endoscopy was found to have high diagnostic performance for BE detection (98% sensitivity and 100% specificity) comparable to standard WLE [52]; nonetheless, since an enriched surveillance population was examined in a tertiary‐care center, the generalizability of this research needs to be further studied. Ultrathin endoscopy was also evaluated for gastric neoplasia detection in 57 patients with superficial gastric neoplasia or undergoing follow‐up endoscopy after ESD, reporting significantly worse performance characteristics than high‐definition WLE [53]. This can be ascribed to the increased difficulty in scope manipulation and the relatively poor imaging quality. As the latest ultrathin models are equipped with better illumination and NBI capability [54, 55], its potential role in the upper GI tract should be further evaluated.

### 2.5 Capsule endoscopy

First approved by the FDA in 2001, capsule endoscopy provides easy and safe access to the GI tract (Fig. 2A). In the last two decades, capsule endoscopy has revolutionized the management of small bowel disease [56]. Most commercially available capsule endoscopes consist of a miniaturized imaging sensor, illumination, and imaging optics, and an internal power supply encased in a disposable enclosure. Data transmission is usually achieved wirelessly *via* radio telemetry or electric‐field propagation [57], and wired data retrieval has also been reported in tethered capsules [58]. Typical imaging specifications of commercially available capsule endoscopy are shown in Table 2.

Given the successful application of capsule endoscopy in the small bowel, there is an increasing interest to investigate its role in other parts of the GI tract. In the upper GI tract, capsule endoscopy is challenging due to the unique anatomy. The average capsule transit time through the esophagus can be as low as about 30 s [59], mandating a fast frame rate to capture high‐quality images; once it enters the stomach, the uncontrolled capsule movement further complicates image acquisition. Using the Pillcam UGI capsule that captures 35 frames per second, capsule endoscopy has been shown to visualize important clinical landmarks of the esophagus; in addition, feasibility to view the entire stomach was demonstrated with patients positioned at different planes and angles on an examination bed in a nurse‐led protocol [59]. Owing to its moderate accuracy (sensitivity 78% and specificity 73% for BE detection in a meta‐analysis of nine studies involving 618 patients by Bhardwaj *et al*.), however, it is not recommended for BE screening [7, 60]. In the lower GI tract, capsule endoscopy has also been found to be useful for detecting additional polyps in patients with incomplete colonoscopy [61].

A major drawback of capsule endoscopy is its lack of active locomotion, which compromises its imaging quality and limits its use in luminal organs. Recent technical efforts have been made to overcome this barrier with internal stabilizing or external control mechanisms [62, 63]. Coupled with an external magnetic steering system, capsule endoscopy has been used to survey more capacious parts of the GI tract [64]. A recent study reported its safe use in 3182 asymptomatic participants to visualize focal lesions in the stomach, suggesting its potential for gastric cancer screening [65]. While in an early stage of development, very recent clinical evidence using a updated magnetically controlled capsule system has shown improved imaging quality and maneuverability [66, 67], and future studies to assess its diagnostic value are warranted.

## 3 Microscopic imaging systems

While macroscopic imaging modalities form the fundamentals of GI endoscopy, the gold standard for cancer diagnosis remains microscopic examination. In the routine practice, this is achieved by acquisition of biopsies followed by standard histology procedures. This process can lead to unnecessary biopsies and related healthcare costs, delay of diagnosis, and loss to follow‐up. As a result, there has been a long‐standing interest in developing *in vivo* endoscopic techniques to facilitate lesion characterization with microscopic or near‐microscopic resolution. In this section, we will review endomicroscopic imaging techniques (Table 4), including commercially available systems such as CLE and volumetric laser endomicroscopy (VLE), and investigative research systems such as the high‐resolution microendoscope (HRME) and capsules based on optical coherence tomography (OCT).

**Table 4:** Microscopic imaging techniques used in the GI tract [97, 138, 139, 143, 144].

Imaging modality	CLE	Endocytoscopy	OCT	HRME
Endoscopic form factor	Endoscope‐ or probe‐based	Endoscope‐ or probe‐based	Probe‐, capsule‐ or balloon‐based	Probe‐based
Source of contrast	Fluorescence	Reflectance	Reflectance	Fluorescence
Contrast agent	Fluorescein	Methylene blue and crystal violet	NA	Proflavine
Targeted clinical features	Extracellular matrix	Cellular architectural morphology	Cellular architectural morphology	Cell nuclei
Resolution	1–3.5 μm	1.7–4.2 μm	~ 10 μm	4.4 μm
Imaging depth	Up to 70 μm	Surface	1–2.5 mm	Surface
FOV	200–300 μm	120–700 μm	Large FOV with pullback	790 μm
Phase of development	Commercially available; extensively evaluated in the entire GI tract	Commercially available; clinically evaluated in the entire GI tract	Commercially available; mostly evaluated in esophagus	Evaluated in the esophagus and colon
Comments on clinical applicability	Compatible with molecular probes; high cost	Compatible with exogenous dyes and advanced imaging such as NBI; high cost	Label‐free and allows large‐area scanning; incompatible with dyes or molecular probe; high cost	Potentially compatible with molecular probes; low cost

### 3.1 Confocal laser endomicroscopy

By illuminating stained tissue with a scanning low‐power laser, CLE (Fig. 2C) can generate fluorescence images of the mucosal layer with micron‐level resolution, providing histologic information similar to standard pathology (Table 4). To image deep epithelial layers with high contrast, confocal scanning is implemented for optical sectioning. Two types of fluorescent contrast agents can be used, including fluorescein that is intravenously administered, and topically applied vital dyes such as acriflavine, tetracycline, or cresyl violet. Fluorescein enhances the contrast of extracellular matrix such as mucosal crypts and villi, as well as vascular structures. Topically applied dyes stain nuclei and thus visualize nuclear morphometry. In clinical practice, fluorescein is FDA‐approved and most widely used without adverse effects [68], and consensus classification criteria referred to as the Miami Classification have been established [69].

Overall, high‐performance characteristics have been achieved using CLE for detection of BE‐related dysplasia (pooled sensitivity and specificity of 89% and 83% in a meta‐analysis of 789 patients) [70], gastric neoplasia (pooled sensitivity and specificity of 81% and 98% in 657 patients) [71], and CRC neoplasms (pooled sensitivity and specificity of 83% and 90% in 376 patients) [72]. Nonetheless, concerns regarding the heterogeneity in performance characteristics and diagnostic yield were raised in recent meta‐analyses [25, 29, 70], indicating the importance of expertise and need for comprehensive training. While providing favorable diagnostic performance in many studies conducted in academic research centers, the routine use of CLE is largely hindered by the significant up‐front investment. In addition, its small FOV can be prone to sampling error for targeted biopsies, especially when operated by novice users to probe small lesions with a stable FOV. Mosaicking in the GI tract to image regions with a larger surface area has been demonstrated in preliminary feasibility studies, but the clinical utility is still limited [73, 74]. Overall, CLE, like many other emerging microscopic modalities, is mostly evaluated in tertiary centers. Without a better understanding of its learning curve and a thorough cost–utility analysis, the practicality for community use is still unknown.

### 3.2 Endocytoscopy

Endocytoscopy is in principle similar to magnification WLE that offers reflectance imaging with up to 150‐fold optical magnification, except that the optical magnification is further improved for imaging at the cellular level (Table 4). Commercially available systems are either endoscope‐based or probe‐based [75], and they support optical magnification of approximately 500‐fold (endoscope‐based) to > 1000‐fold (probe‐based). To enhance mucosal surface contrast under white light illumination, methylene blue or its combination with crystal violet has been found useful for visualizing nuclear and glandular patterns [76].

Commercial endocytoscopy systems have been shown to allow for cellular‐level characterization of GI lesions [77, 78, 79]. Recent studies investigated the effectiveness of endocytoscopy to tackle challenging tasks such as diagnosing adenomatous diminutive polyps and low‐grade adenoma in the colon, reporting high diagnostic performance (96.8% accuracy in 39 patients and 86.4% accuracy in 573 patients, respectively) [80, 81]. To facilitate its broader application, efforts have been made to develop classification systems for standardized clinical interpretation [82]. Different from fluorescence‐based CLE, endocytoscopy provides a reflectance modality to investigate suspicious lesions at ultrahigh resolution. In addition to exogenous dyes, it offers compatibility with virtual CE such as NBI. While clinical evidence from large‐scale RCTs is still unavailable, its further evaluation is supported by initial studies reporting performance characteristics comparable to CLE.

### 3.3 Optical coherence tomography

Using a low‐coherence light source to probe tissue reflectance at varied transverse locations, OCT generates depth‐resolved, near‐microscopic images with axial resolution of about 10 µm and lateral resolution of approximately 30 µm (Table 4). Since OCT images are acquired *via* a single‐mode fiber, it is inherently compatible with endoscopic imaging of luminal organs including the GI tract and cardiovascular systems. To access lesions in the GI tract, OCT systems of different form factors have been developed, including probe‐ and balloon‐based OCT that can pass through an endoscope working channel, and a capsule‐based design that can be operated in the primary care setting (Table 4). Using OCT in different forms, depth‐resolved and high‐resolution imaging has been reported in Barrett's esophagus, stomach, small intestine, and colon polyps.

In 2013, an OCT‐based imaging system became commercially available for esophageal imaging, known as the VLE (NvisionVLE, NinePoint Medical, Bedford, MA, USA; Fig. 2B). In the VLE, a balloon catheter is used to center the OCT probe, enabling stable circumferential scanning of a 6 cm segment of esophagus with a 3 mm imaging depth in 90 seconds. Since its introduction, studies have been conducted to establish image interpretation criteria, reporting a diagnostic accuracy of 87% for BE‐related dysplasia in a pilot study of 27 patients [83]. Moreover, artificial intelligence (AI)‐based algorithms are developed and clinical evaluation is underway in a multicenter RCT [84]. Another important extension of the VLE technology is the introduction of laser marking in 2016, enabling a targeted biopsy protocol shown to lead to a higher yield of dysplasia in BE compared with the standard Seattle protocol (33.7% in 106 patients vs. 19.6% in 95 patients) [85]. While the majority of OCT studies focused on the esophagus, successful imaging of the colon has been reported in case studies or case series [86, 87]. Unlike the tubular esophagus, good tissue contact with the balloon catheter to ensure VLE imaging quality can be more difficult in the colon.

Thanks to the scanning and pullback mechanism, OCT systems can generate depth‐resolved and near‐microscopic maps of a long esophageal segment, and cross‐sectional visualization of multiple histologic layers can provide important information for detecting disease progression beneath the superficial surface. Combined with a laser marking system, VLE is uniquely poised to reduce sampling errors, a major limitation for other endomicroscopic modalities. Nonetheless, commercial VLEs are costly and clinical interpretation of reflectance‐based volumetric images remains a challenging process, even though computer‐aided systems are under evaluation. In addition, as OCT captures tissue reflectance, it precludes the use of specific contrast agents and molecular probes.

Many recent technical innovations in endoscopic OCT have embraced a capsule enclosure, allowing for miniature optomechanical components to be included at distal end of the probe and improving imaging quality and contrast. Spatial resolution, for example, can be improved with a miniaturized actuation system to minimize rotational distortion [88], or using a shorter wavelength to enable ultrahigh‐resolution OCT [89]. OCT‐based endoscopy also benefited from an increased scanning speed, allowing for ultra‐fast OCT angiography to visualize subsurface microvasculature in the GI tract [90]; briefly, circumferential OCT images are acquired at 400 frames per second, and decorrelation between sequential frames is calculated to highlight circulating erythrocytes and thus the 3D microvasculature. In addition to circumferential imaging of luminal organs, a piezoelectric probe was also developed to enable forward‐viewing OCT imaging of colorectal polyps [91]. Enabled by different scanning mechanisms such as microelectromechanical system and piezoelectric transducers to form a 2D sampling pattern, forward‐viewing OCT probes can generate high‐resolution and label‐free images with an intuitive view similar to standard endoscopy.

### 3.4 High‐resolution microendoscope

While offering superior resolution compared to conventional endoscopes, the high cost of the above‐mentioned endomicroscopic modalities limits their clinical use in tertiary medical centers. As an alternative shown in Fig. 2D, the HRME is a low‐cost ($1500), fiber‐optic fluorescence microscope that can image nuclear morphology at subcellular resolution [92]. In pilot clinical trials, the clinical utility of HRME was demonstrated for accurate colon polyp characterization using proflavine as an inexpensive and topically applied dye for vital staining [93, 94]; in a prospective single‐center trial, HRME was shown to have a high accuracy and specificity of 94% and 95% for detecting adenomatous and neoplastic polyps during routine screening or surveillance colonoscopy of 94 patients [95]. In the upper GI tract, HRME has also shown value for detection of BE‐related dysplasia and ESCC [96, 97]. In a prospective trial of 147 high‐risk patients, adjunctive HRME following Lugol's CE was shown to increase the specificity from 48% to 88% for ESCC detection while achieving a sensitivity of 91% [98].

To further facilitate dissemination of this high‐resolution yet inexpensive technology, quantitative and automated diagnostic algorithms have been developed and prospectively validated [97, 99, 100]. Recent development focused on further improvement of its axial resolution with optical sectioning techniques [101, 102]. Compared with commercial CLE, HRME offers a low‐cost alternative to visualize important histologic features in community and resource‐constrained settings.

### 3.5 Other *in vivo* microscopy technologies

Various emerging technologies at earlier stages of development have also been reported in animal models or case studies. Multiphoton endomicroscopy, for example, is a novel technology that has gained attention as a high‐resolution and label‐free imaging modality. Taking advantage of near‐infrared laser excitation and endogenous autofluorescence, recent studies in rodent animal models reported deeper penetration (120 µm) than CLE without administration of contrast agent [103, 104].

Spectrally encoded confocal endomicroscopy (SECM) is another fiber‐optic imaging modality that can image tissue surface reflectance using a single‐mode fiber coupled with a diffraction grating that encodes tissue locations with distinct wavelengths. Integrated with a rotary junction and a pullback mechanism, high‐speed line scanning of large areas can be achieved [105] Like OCT‐based capsules, a tethered SECM capsule was shown feasible for imaging of the esophagus [106].

In addition to imaging modalities that offer direct visualization, spectroscopic probes have also been developed to study the subcellular architecture and molecular constituents of GI lesions. Briefly, endoscopic spectroscopy measures the spectral composition of backscattered light from tissue under varied illumination wavelengths and geometries, exploiting three major types of light–tissue interactions—elastic scattering, absorption, and inelastic scattering. Elastic scattering, as the dominant type of scattering, occurs without wavelength change and is sensitive to alterations in cell and tissue architecture. In addition, tissue absorption, mostly from hemoglobin, lipids, and water, also contributes to elastic scattering spectra in the visible and near‐infrared spectral regions. In comparison, inelastic (or Raman) scattering with wavelength shifts is relatively weaker, but can be employed to characterize chemical fingerprints of specific molecular components. A broad range of spectroscopic techniques, including diffuse reflectance spectroscopy and Raman spectroscopy, have been clinically evaluated with varied results in the GI tract [107, 108, 109, 110]. Compared to imaging modalities, spectroscopic techniques provide label‐free, quantitative, and objective measurements of GI mucosa. Nonetheless, its clinical interpretability is limited by the complexity of high‐dimensional data from a point‐to‐point FOV. With recent studies reporting technical advances such as novel fiber design and nanoparticles [111, 112], further evaluation in larger clinical trials is warranted for this broad range of technologies.

## 4 Molecular imaging

Most existing modalities detect early cancer lesions based on the presence of macroscopic or microscopic morphological changes. By studying the altered biomolecular cascades that may precede architectural changes, molecular imaging is an emerging field in modern oncology that has the potential to detect GI lesions at earlier stages with higher specificity than morphologic alterations. Driven by genetic and molecular profiling of tumors, specific biomarkers can be targeted using a wide range of molecular probes, including antibodies, peptides, nanoparticles, aptamers, and affibodies [113]. Since molecular probes are typically fluorescently labeled, they are mostly used in conjunction with fluorescence imaging modalities such as CLE or widefield fluorescence endoscopes. *Ex vivo* near‐infrared imaging has also been employed to suppress confounding tissue autofluorescence.

While most studies are focused on proof of concept in rodent animal models, recent studies have demonstrated potential for *ex vivo* and *in vivo* applications in human trials. In Barrett's esophagus, lectin‐based probes were found useful for differentiating dysplastic from benign lesions in *ex vivo* human specimens [114, 115]. Sturm *et al*. [116] developed a peptide that binds specifically to BE‐related dysplasia and demonstrated its safe *in vivo* application using the CLE. A similar study was conducted for CLE‐based targeted imaging of EGFR in the colon [117]. Thanks to its specific binding, molecular probes can also been used with widefield fluorescence endoscopes for detection of flat colon lesions [118, 119].

Molecular probes in combination with fluorescence endoscopic imaging provide novel routes to targeted imaging with high specificity, addressing a key limitation of many widefield endoscopic modalities. Another advantage of this approach, as demonstrated in animal models, is the safe use for longitudinal studies without tissue removal [120]. Recent successful translation of this approach into human trials shows promising results for functional characterization of GI lesions, thereby holding potential for personalized treatment recommendations. Future studies in this intrinsically multidisciplinary field will benefit from collective efforts to develop novel probes and imaging devices, while strictly monitoring safety during clinical use.

## 5 Machine learning for computer‐aided detection and diagnosis

**Fig. 3:**
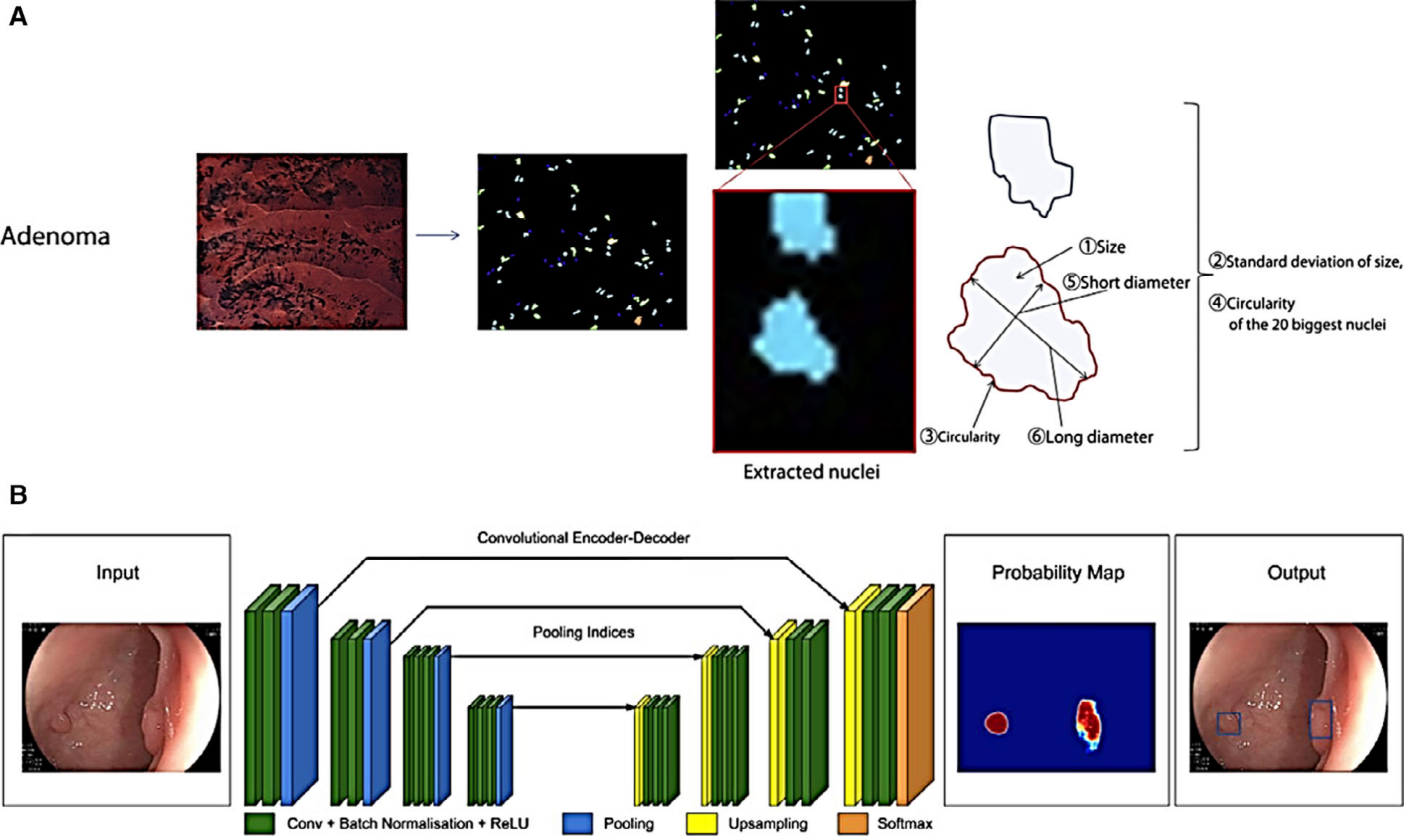
Examples of computer‐aided algorithms for detection and diagnosis of colorectal polyps. (A) In microscopic images of polyps collected with endocytoscopy, clinically inspired nuclear morphology features were extracted and quantified for diagnosing advanced histology. (B) A data‐driven algorithm is trained using a convolutional neural network to highlight adenomatous CRC polyps in WLE images. Figure 3A reproduced from [134] with permission from Elsevier. Figure 3Breproduced from [125] with permission from BMJ Publishing Group Ltd.

With the advent of various novel endoscopic techniques, objective and confident interpretation of multiscale and multidimensional clinical data is a crucial, yet cumbersome and challenging task. In the meantime, the abundance of large‐volume imaging data presents numerous opportunities to employ machine learning to assist novice endoscopists and experts alike. From an algorithm development perspective, two approaches have been used to facilitate clinical decision making. First, in classical machine‐learning methods, as illustrated in Fig. 3A, a broad range of features can be explored and extrapolated, such as shape, texture, color, and clinically inspired features [121]. After feature extraction, a classifier can be developed for computer‐aided diagnosis (CADx). Second, recent implementation of deep learning algorithms has been shown to contribute to more sophisticated examination of the feature space, while enabling accelerated processing of large datasets in real time (Fig. 3B). Compared with classical machine‐learning approaches, deep learning scales more effectively with large datasets and thus is well suited for processing a series of images with high dimensions (e.g. multimodal images); in addition, it also alleviates the burden of complicated feature engineering. Nonetheless, a large and standardized dataset is usually required for effective training of deep learning algorithms, and their clinical interpretability is relatively poor. In this section, we discuss recent advances of machine‐learning approaches to perform specific clinical tasks.

In terms of clinical output, algorithms are generally trained to perform either computer‐aided detection (CADe) such as lesion detection using macroscopic modalities, or CADx based on microscopic or high‐definition widefield imaging. To date, CADe algorithms have been most extensively applied to improve automated colorectal polyp detection [122]. Initial studies in the field focused on polyp detection rate as the primary performance measure, and recent advancement in computing hardware and algorithms has significantly accelerated image processing for real‐time and video‐rate applications [123, 124]. Very recently, in a prospective RCT involving 1057 patients, Wang *et al*. reported significantly increased ADR in WLE images using an AI‐based system than standard colonoscopy (29.1% vs. 20.3%) [125]. Of note, the ADR improvement is attributed to detection of more diminutive polyps. Diagnostic algorithms (CADx) to differentiate adenomatous from benign colorectal polyps were also developed in retrospective studies [126], with a recent study reporting a high accuracy of 94% for diagnosing adenomatous diminutive polyps in unaltered NBI videos [127]. Similarly, excellent accuracies were reported to diagnose neoplastic lesions in the esophagus and stomach in widefield modalities, including WLE [128], NBI [129], or their combination [130]. When integrated with standard WLE or advanced widefield modalities such as NBI, these diagnostic algorithms can facilitate more quantitative and objective interpretation of clinical data; for novice users, such algorithms can provide critical decision support that can reduce the learning curve and improve the diagnostic accuracy. Microscope imaging modalities, including CLE, HRME, endocytoscopy, and VLE, have also benefited from automated algorithms [99, 100, 131, 132, 133, 134, 135].

It is evident that machine learning is playing an increasingly significant role in the present field of GI endoscopy. While most algorithms are developed and evaluated retrospectively, prospective studies with real‐time and low‐latency algorithms are emerging. With the integration of AI systems in recently launched commercial systems, including EndoBrain in endocytoscopy systems (Olympus) and Intelligent real‐time image segmentation in the NvisionVLE imaging system (NinePoint Medical), it is hoped that machine learning will contribute to palpable changes in routine practice in the coming years. As machine learning in GI endoscopy is experiencing rapid progress, there remain several barriers for clinical implementation. First, it is of crucial importance to validate the generalizability of computer‐assisted algorithms, especially when images are acquired by users in varied settings. Second, for users to make best use of computer‐aided decision support, interpretability and training are also important factors when designing the algorithm architecture. Finally, the intricate nature of computational algorithms can raise new ethical and regulatory concerns, and their role during practical use will ultimately depend on acceptance by the healthcare community.

## 6 Conclusion and outlook

The past few decades have witnessed substantial evolution of optical endoscopy techniques and their continuous clinical translation to enable *in vivo* characterization of the GI mucosa with unpreceded imaging contrast, resolution, depth, and speed. The multidimensional, volumetric, and real‐time imaging data open new opportunities for improved detection of GI pathology, potentially leading to important shifts in diagnostic and therapeutic algorithms. Moreover, novel molecular probe and machine‐learning methods are under clinical evaluation, showing great promises to improve detection accuracy and reliability. Taken together, the constantly evolving landscape of modern GI endoscopy presents numerous opportunities and demands a multidisciplinary and collaborative effort in the academic and healthcare community. In the meantime, with academic‐industrial partnerships playing an increasingly important role in prototype development and commercialization, close integration of academia and industry is also called for to accelerate technology translation, standardization, and dissemination.

The rapid advent of novel techniques also presents new challenges. While offering direct visualization of GI lesions, acquisition and interpretation of clinical data using optical imaging techniques require adequate training and expertise. Since a large proportion of these clinical studies are conducted in research or academic centers, it is of paramount importance to standardize their clinical use and validate diagnostic performance in varied clinical settings. Once validated in large‐scale studies, there are challenges to balance increased cost, learning curves, and user resistance to new technology. Therefore, in addition to technical performance metrics, clinical barriers for technology adoption should be assessed when placing novel devices into the hands of practitioners. Nonetheless, clinical adoption of novel technologies has taken place rapidly; for example, the use of capsule endoscopy for small bowel disease and NBI for colorectal polyp characterization shows that wide scale adoption is possible in short time frames. With an ever‐increasing amount of technical innovations and clinical data, we expect that novel optical imaging technologies will lead to significant changes in the clinical workflow in the coming decade, thus improving outcomes for a large at‐risk population through more accurate and less invasive endoscopic imaging.

## Conflict of interest

The authors declare no conflict of interest.

## Author contributions

YT, SA, and RR‐K wrote the paper.
